# Simultaneous quantification of fentanyl, sufentanil, cefazolin, doxapram and keto‐doxapram in plasma using liquid chromatography–tandem mass spectrometry

**DOI:** 10.1002/bmc.4290

**Published:** 2018-06-12

**Authors:** Robert B. Flint, Soma Bahmany, Bart C. H. van der Nagel, Birgit C. P. Koch

**Affiliations:** ^1^ Erasmus University Medical Center Department of Pharmacy Rotterdam the Netherlands; ^2^ Erasmus University Medical Center—Sophia Department of Pediatrics, Division of Neonatology Rotterdam the Netherlands; ^3^ Department of Pharmacy and Radboud Institute of Health Sciences Nijmegen The Netherlands

**Keywords:** cefazolin, doxapram, fentanyl, sufentanil, UPLC–MS/MS

## Abstract

A simple and specific UPLC–MS/MS method was developed and validated for simultaneous quantification of fentanyl, sufentanil, cefazolin, doxapram and its active metabolite keto‐doxapram. The internal standard was fentanyl‐d5 for all analytes. Chromatographic separation was achieved with a reversed‐phase Acquity UPLC HSS T3 column with a run‐time of only 5.0 min per injected sample. Gradient elution was performed with a mobile phase consisting of ammonium acetate or formic acid in Milli‐Q ultrapure water or in methanol with a total flow rate of 0.4 mL min^−1^. A plasma volume of only 50 μL was required to achieve adequate accuracy and precision. Calibration curves of all five analytes were linear. All analytes were stable for at least 48 h in the autosampler. The method was validated according to US Food and Drug Administration guidelines. This method allows quantification of fentanyl, sufentanil, cefazolin, doxapram and keto‐doxapram, which is useful for research as well as therapeutic drug monitoring, if applicable. The strength of this method is the combination of a small sample volume, a short run‐time, a deuterated internal standard, an easy sample preparation method and the ability to simultaneously quantify all analytes in one run.

**Abbreviations**
TDM: therapeutic drug monitoring

## 1 INTRODUCTION

One of the most important issues in peri‐operative and intensive care medicine is the establishment of an individual antibiotic and analgosedation drug profile for each patient with respect to the clinical situation, together with support of vital functions. In general, the analgesic and sedation dose regime will be adjusted to the clinical situation of each individual patient to shorten the duration of therapy and to reduce morbidity (Chanques et al., 2006). Large knowledge gaps exist with respect to the optimal drug therapy and covariates that determine the effect and safety, especially in neonates and infants, and well‐designed trials are required (Coppini et al., 2016).

For certain drugs therapeutic drug monitoring (TDM) has been proven valuable for monitoring of drug effects, dosage regimes and physiological changes, and when appropriate, adapting the medical care to each patient in the intensive care unit. Despite the fact that many drugs are still being dosed on clinical response, the continuously expanding assortment of analytical methods improves drug safety and individual patient treatment (Touw et al., 2005). For research as well as for TDM, more assays are neeeded with a minimal sample volume, a short run time, quick and easy sample preparation, and simultaneous measurement of multiple analytes. Simultaneous quantification in one assay allows quantification of multiple analytes in one sample without requiring extra sample volume, and allows to samples to be run containing different drugs efficiently in one assay‐run.

Evidence is sparse on the use of fentanyl, sufentanil, cefazolin and doxapram for certain pediatric age‐ranges and indications. Sufentanil, fentanyl and cefazolin are part of peri‐operative treatments for children. Furthermore, sufentanil and fentanyl are synthetic opioid analgesics widely used in clinical anesthesia and analgesia (Mather, 1983; Pacifici, 2015). Cefazolin is a first‐generation cephalosporin beta‐lactam antibiotic used for treatment of sepsis or life‐threatening infections (McWhinney et al., 2010), where adequate individual dosing may be lifesaving. Doxapram has not been investigated sufficiently in children, despite its frequent and promising use in neonatal intensive care for treatment of apnea of prematurity (Flint et al., 2017, Flint et al., 2017; Pacifici, 2015; Prins et al., 2013).

This assay will aid future research to close the knowledge gaps on these four drugs, but may also be used for TDM if a target concentration range can be defined. As these four drugs are commonly prescribed and combined, we aimed to develop and validate a quick and easy analytical method for simultaneous quantification of fentanyl, sufentanil, cefazolin, doxapram and its active metabolite keto‐doxapram in human plasma by ultra‐performance liquid chromatography–electrospray ionization–tandem mass spectrometry (UPLC–MS/MS). We optimized the sensitivity of the assay so to minimize the required sample volume, which allows measurement of small volume samples, even from premature born infants.

## 2 MATERIALS AND METHODS

### 2.1 Chemicals and reagents

Fentanyl, fentanyl‐d5 and sufentanil were purchased from Sigma Aldrich (Zwijndrecht, the Netherlands). Cefazolin was obtained from Santa Cruz Biotechnology (Heidelberg, Germany), doxapram from Selleckchem (Munich, Germany) and keto‐doxapram from Tractus (London, UK). Ammonium acetate was obtained from Sigma Aldrich (Zwijndrecht, the Netherlands). Methanol, acetonitrile and formic acid were purchased from Biosolve BV (Valkenswaard, the Netherlands). All reagents were LC–MS grade, which means at least 99% purity. Water was purified by using a MilliPore Advantage A10 system. Human drug‐free plasma was obtained from the blood donation center (Sanquin, Rotterdam, the Netherlands).

### 2.2 Stock solutions, calibration standards, quality control samples and internal standard

Stock solutions of doxapram and keto‐doxapram were prepared at a concentration of 500 mg L^−1^ using methanol. The following substance stock concentrations in methanol were prepared: sufentanil at a concentration of 20 mg L^−1^; fentanyl at a concentration of 2 mg L^−1^; and cefazolin at a concentration of 5,000mg L^−1^ using Milli‐Q water. For each analyte two separate stock solutions were made with the same concentration, for calibration of standard samples and for quality control samples. Stock solutions were stored at −20°C, except the stock solution of cefazolin which was stored at 2–8°C. The calibration standard 8 and quality control (QC) high were made from the stock solutions with drug‐free human plasma. Calibration standards 1–7 and the lower limit of quantification (LLOQ) standard were prepared by serial dilution of calibration standard 8 with human plasma. QC medium and QC high samples were prepared the same way, using the other stock solution (QC high), which was diluted with human plasma. The concentrations of all calibration standards are given in Table 1 and the concentrations of the quality controls are given in Table 2. Calibration standards and quality control samples were stored as 50 μL portions in 1.5 mL Eppendorf tubes at −80°C prior to analysis. The internal standard was fentanyl‐d5, which was dissolved in a mixture of acetonitrile and methanol 1:1 at a concentration of 10 μg L^−1^. The internal standard working solution was stored at −20°C.

**Table 1:** Concentrations of all calibration standards and lower limit of quantitation (LLOQ) standard

Analyte	LLOQ (μg L^−1^)	S1 (μg L^−1^)	S2 (μg L^−1^)	S3 (μg L^−1^)	S4 (μg L^−1^)	S5 (μg L^−1^)	S6 (μg L^−1^)	S7 (μg L^−1^)	S8 (μg L^−1^)
Fentanyl	0.10	0.10	0.50	1.0	2.5	4.0	5.0	8.0	10
Sufentanil	0.25	1.0	5.0	10	25	40	50	80	100
Cefazolin	1,000	1,000	5,000	10,000	25,000	75,000	80,000	90,000	100,000
Doxapram	50	100	500	1,000	2,500	4,000	5,000	8,000	10,000
Keto‐doxapram	50	50	100	250	500	1,000	2,000	4,000	5,000

S, Calibration standard.

**Table 2:** Concentrations of all quality controls

Analyte	QC low (μg L^−1^)	QC medium (μg L^−1^)	QC high (μg L^−1^)
Fentanyl	0.5	2.5	7.5
Sufentanil	2.0	10	30
Cefazolin	4,000	25,000	70,000
Doxapram	400	2,500	7,000
Keto‐doxapram	150	850	3,000

QC, Quality control.

### 2.3 Sample preparation

A mixture of acetonitrile and methanol, containing 10 μg L^−1^ fentanyl‐d5 (the internal standard solution), was used for protein precipitation. A 50 μL aliquot of the calibration standards, quality control samples, blanks and patient samples were thawed at least half an hour prior to preparation. Then plasma proteins were precipitated by adding 200 μL of the internal standard solution. Subsequently, the samples were vortexed for about 10 s. After vortexing, the precipitant was separated by centrifugation for 5 min at 16,000 ***g***. A 100 μL aliquot of each supernatant was transferred into an autosampler insert vial (VWR, Amsterdam, the Netherlands) and diluted by adding 400 μL of mobile phase A. The autosampler vials were mixed using the vortex for 10 s. For cefazolin, doxapram and keto‐doxapram 1 μL was injected into the UPLC. For fentanyl and sufentanil, 10 μL was injected into the system because of the lower therapeutic range of these compounds (see Table 2).

### 2.4 Instrumentation

A Dionex Ultimate UPLC system consisting of an Ultimate 3,000 RS UPLC pump, an Ultimate 3,000 RS autosampler and an Ultimate 3,000 RS Column Compartment was used as the equipment. The UPLC was connected to a Thermo TSQ Vantage triple quadrupole MS with HESI probe (Thermo Scientific, Waltman, MA, USA). The software programs Chromeleon (version 6.8, Dionex, Thermo Scientific), Xcalibur (version 2.1, Thermo Scientific) and LCquan (version 2.6, Thermo Scientific) were used to control the system and analyze the data.

### 2.5 UPLC conditions

Chromatographic separation, based on affinity of the analytes with the nonpolar stationary phase, was achieved with a reversed‐phase UPLC Acquity BEH C_18_ column, 1.7 μm, 2.1 × 100 mm (Waters, Milford, USA). Gradient elution was performed with a mobile phase consisting of 1 mL of a 154 mg/L solution of ammonium acetate in formic acid (99%) in 1 L of Milli‐Q ultrapure water (eluent A) and 1 mL of the same solution in 1 L of methanol (eluent B). Before the analysis, the system was equilibrated at the starting conditions of 75% eluent A and 25% eluent B until pressure was stable. The multistep gradient was as follows: from 0 to 0.6 min, eluent B was increased from 25 to 48%; from 0.6 to 1.5 min, eluent A decreased to 35% and B was increased to 65%; from 1.6 to 2.8 min, eluent B was kept stable at 100% and 0% eluent A; from 3.0 to 5.0 min, eluent A was inceased to 75% and B was decreased to 25%. The run ended at 5.0 min at starting conditions. Temperature for the column oven was set at 50°C and for the autosampler at 15°C.

The separation was performed by gradient elution using mobile phase A (1 mL of 2 m ammonium acetate in formic acid 99%), in 1 L Milli‐Q water and mobile phase B (1 mL of 2 m ammonium acetate in formic acid 99%), in 1 L methanol with a total flow rate of 0.4 mL min^−1^. Mobile phase B was kept at 25% from 0.0 to 0.6 min, then at 48%, from 0.6 to 1.5 min mobile phase B at 65%, then from 1.6 to 2.8 min at 100%, from 3.0 to 5.0 min mobile phase B was kept at 25%. The run ended at 5.0 min at starting conditions. Temperature for the column oven was set at 50°C and for the autosampler at 15°C.

### 2.6 MS/MS conditions

MS/MS detection was performed in positive mode using selected reaction monitoring (SRM) with electrospray ionization. To optimize the MS/MS parameters to detect the most intense signal of each analyte, solutions of 1 mg L^−1^ were directly infused in methanol by addition of the mobile phase (75% mobile phase A and 25% mobile phase B) from the LC at a flow rate of 0.4 mL min^−1^. The MS/MS instrument was operated with a capillary spray voltage of 3 kV, vaporizer temperature at 375°C, capillary temperature at 250°C, sheath gas pressure at 50 (arbitrary units), auxiliary nitrogen gas pressure at 20 (arbitrary units) and collision gas pressure at 1.5 mTorr. Specific parameters for each compound are given in Table 3.

**Table 3:** MS/MS settings

Analyte	Parent ion (*m*/*z*)	Product ion (*m*/*z*)	Collision energy (V)	S‐Lens (V)
Fentanyl	337.4	188.2	22	124
Fentanyl‐d5	342.4	188.2	22	124
Sufentanil	387.3	238.2	18	124
Cefazolin	455.1	323.0	10	80
Doxapram	379.3	128.1	55	135
Keto‐doxapram	393.2	214.1	26	150

### 2.7 Assay validation

Validation of the method was performed according to the US Food and Drug Administration (2003) guidelines for bioanalytical methods. The following validation parameters were investigated: linearity, LLOQ and upper limit of quantification (ULOQ), accuracy, repeatability, reproducibility, stability and matrix effect.

#### 2.7.1 Linearity

To investigate the linearity of the method, a blank sample (without internal standard), a zero sample (blank with internal standard) and eight calibration standards in duplicate were prepared and analyzed. Calibration curves were generated by plotting the theoretical standard concentration vs the ratio of the standard peak area to the internal standard area. The determination coefficient (*R* ^2^) should be at least 0.9950. The relative standard deviation (RSD) of the calculated concentrations of the standard concentrations was required to be <15%, except at the LLOQ, where it should not deviate by more than 20%. It was decided to apply weighting 1/*x*, which means that standards with the lowest concentrations are more important for the calibration line than standards with highest concentrations (Saar et al., 2010). The calibration curves were formed using the peak area ratios for the analytes and their corresponding internal standard (response) vs the concentrations applying linear least square regression with a weighing factor of 1/*x* and excluding of the origin.

#### 2.7.2 LLOQ and ULOQ

The LLOQ was measured by analyzing the LLOQ standard six times in a row. Mean and standard deviation of the response ratios of the six samples were measured. The response of the analyte should be at least 5 times the response compared with the response of the blank. Precision and accuracy were calculated and should be ≤20% and the accuracy should be between 80 and 120%. The highest standard of the calibration curve was used as the ULOQ.

#### 2.7.3 Accuracy

Accuracy was measured by measuring three concentrations (QC‐H, QC‐M and QC‐L) six times on the same day. The percentage difference between the measured concentration and the theoretical concentration, known as the relative standard deviation (RSD), was required to be <15%.

#### 2.7.4 Repeatability and reproducibility

The repeatability was tested by analyzing three QC levels six times on the same day. The reproducibility was tested by analyzing three concentrations in duplicate on six different days. The requirement for both parameters was an RSD <15%.

#### 2.7.5 Stability

Autosampler stability was determined by storing QC samples (*n* = 2 per concentration) after sample preparation in the autosampler for 24, 48, 72 and 120 h. Response ratios were measured and compared with response ratios of samples kept at −80°C prior to preparation. After sample preparation, samples were directly analyzed. Recovery was required to be between 90 and 110%.

#### 2.7.6 Matrix effect

It is important to measure matrix effects and absolute recoveries in the development of an LC–MS/MS method since ion suppression and ion enhancement effects can be expected owing to interferences by matrix compounds, stable‐isotope‐labeled internal standards and co‐eluting compounds (Van Eeckhaut et al., 2009). In order to check whether the precision, the reproducibility and the stability of the concentration‐signal ratio are affected by interference of the matrix analytes, the method described by Matuszewski et al. (2003) was used. Five different lots of human plasma were used. To two concentration levels (QC low and QC high, both in duplicate), the analytes were added before and after extraction, which served to calculate the recovery. Also, a set of six academic samples was evaluated with only Milli‐Q ultrapure water instead of plasma. Matrix effects were calculated as follows:

peak area of analyte spiked after extraction/peak area of analyte prepared in Milli‐Q ultrapure water × 100%.

The process efficiency was calculated as the percentage ratio of the area of the analytes spiked before extraction and the ones prepared in Milli‐Q ultrapure water. The mean and RSD were calculated of matrix effects, process efficiency and recovery. In the ideal situation, the mean matrix effects, process efficiency and recovery are between 80 and 120%, and the RSD of both parameters is ≤15%. Furthermore, for each analyte, the internal standard normalized matrix effect should also be calculated by dividing the matrix effect of the analyte by the matrix effect of the IS. The RSD of the internal standard‐normalized matrix effect calculated from the different lots of matrix should not be greater than 15%.

### 2.8 Clinical application

The method was developed for the analysis of plasma samples from a pharmacokinetic study and may also be used for TDM, if this can be proven clinically valuable. For the validation of the assay for clinical practice, clinical application and research purposes the method was applied to quantify doxapram, keto‐doxapram and fentanyl in plasma of preterm infants participating in a clinical study. The Erasmus Medical Center ethics review board approved the protocol and written informed consent from parents/legal guardians was obtained prior to study initiation (MEC‐2014‐067, ClinicalTrials.gov by NCT02421068). This observational prospective multicenter study was performed between September 2014 and June 2017 at the Departments of Neonatology of the Radboud University Medical Centre in Nijmegen, Maastricht University Medical Centre in Maastricht, Maxima Medical Centre in Veldhoven and Sophia Children's Hospital in Rotterdam. Neonates routinely received doxapram (Dopram®, Manage, Belgium) for treatment of apnea of prematurity starting with a loading dose of 2.5 mg kg^−1^ bodyweight in 15 min, followed by a maintenance starting dose of 2.0 mg kg^−1^ h^−1^, either by continuous intravenous infusion or continuous gastro‐enteral administration. Fentanyl (Bipharma, Almere, the Netherlands) was indicated for comfort as an intravenous continuous infusion of 0.5–2.0 μg kg^−1^ h^−1^ or as a bolus injection of 0.5–3.0 μg kg^−1^.

## 3 RESULTS

### 3.1 Linearity

**Figure 1:**
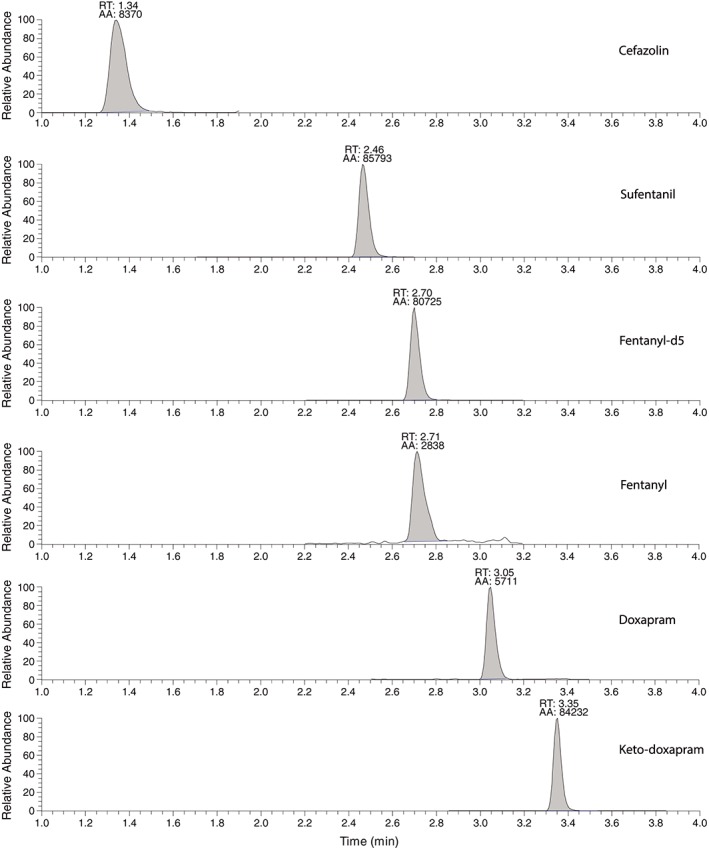
Ion chromatograms of all analytes and internal standard in lowest concentration calibration standard 1 (see Table 1). For the ion chromatograms of sufentanil and fentanyl, 10 μL was injected, and for cefazolin, doxapram and keto‐doxapram the injection volume was 1 μL. RT, Retention time; AA, automatic integrated area

Linearity was achieved for each analyte in the range between the LLOQ and the ULOQ (Table 2), with all RSDs to be <15% and the determination coefficient (*r* ^2^) to be at least 0.995. The calibration curves showed that a regression with a weighting factor of 1/*x* best described the dataset over the range for all analytes. Figure 1 shows the ion chromatograms obtained after the analysis of the lowest plasma calibrator standard for all the analytes, and the corresponding retention times of each analyte (see Table 1).

### 3.2 LLOQ and ULOQ

The results for the LLOQ for cefazolin, keto‐doxapram and fentanyl did not meet the initial requirements. Therefore, the LLOQ for these analytes was set to calibration standard 1, which was acceptable. The results of determination of LLOQ and ULOQ are shown in Table 4.

**Table 4:** Validation results (*n* = 6)

Analyte	QC	Accuracy, RSD (%)	Repeatability (within‐run precision)			Reproducibility (between‐run precision)			LLOQ	ULOQ
			Mean (μg L^−1^)	SD (μg L^−1^)	RSD (%)	Mean (μg L^−1^)	SD (μg L^−1^)	RSD (%)	(μg L^−1^)	(μg L^−1^)
Fentanyl	L	−2.7	0.46	0.01	1.5	0.51	0.02	3.9	0.10	10.0
	M	−2.7	2.1	0.05	2.4	2.00	0.03	1.5		
	H	−3.4	6.8	0.09	1.3	6.82	0.15	2.2		
Sufentanil	L	3.0	5.7	0.06	1.0	5.60	0.14	2.5	0.25	50.0
	M	−0.2	26.7	0.44	1.7	25.5	0.56	2.2		
	H	−1.8	53.1	1.03	1.9	52.5	1.47	2.8		
Cefazolin	L	8.0	6.6	0.12	1.7	6.57	0.23	3.5	1,000	100,000
	M	0.4	32.1	0.39	1.2	30.9	0.34	1.1		
	H	0.9	101.6	1.48	1.5	104.2	1.25	1.2		
Doxapram	L	3.2	0.42	0.01	1.4	0.43	0.02	4.7	50	4,500
	M	3.0	2.15	0.04	1.9	2.22	0.02	0.9		
	H	−1.2	3.46	0.06	1.8	3.57	0.05	1.4		
Keto‐doxapram	L	−4.8	0.16	0.00	1.5	0.18	0.01	5.7	50	5,000
	M	3.2	0.88	0.02	2.1	0.77	0.02	2.6		
	H	1.7	3.43	0.08	2.4	3.43	0.12	3.5		

QC, Quality control; L, low; M, medium; H, high; SD, standard deviation; RSD, relative standard deviation; ULOQ, upper limit of quantification.

### 3.3 Accuracy, repeatability and reproducibility

The RSD of accuracy, repeatability and reproducibility data were within the requirement of an RSD <15% (Table 4).

### 3.4 Stability

Except for cefazolin and keto‐doxapram, the recovery of all QCs was between 90 and 110%, indicating that they were stable for at least 120 h when stored in the autosampler at 15°C. Cefazolin was only stable for 72 h and keto‐doxapram only for 48 h.

### 3.5 Matrix effect

Matrix effects and absolute recoveries in the development of the LC–MS/MS method are shown in Table 5. The method described by Matuszewski et al. (2003) showed that fentanyl, sufentanil, cefazolin, doxapram and keto‐doxapram experienced neither matrix effect nor an effect from the sample preparation. A good recovery was achieved for all analytes.

**Table 5:** Matrix effect, recovery and process efficiency

Analyte	Matrix effect, mean (%)	Recovery, mean (%)	Process efficiency, mean (%)
Fentanyl	113.3	102.2	115.9
Sufentanil	108.8	93.5	101.7
Cefazolin	108.0	90.1	97.4
Doxapram	111.3	92.5	102.9
Keto‐doxapram	99.9	99.2	99.1

### 3.6 Clinical application

A total of 618 samples were collected from a pediatric cohort of preterm infants (*n* = 157), consisting of 92 infants who received fentanyl and 65 infants with doxapram. Eleven samples were collected from six patients from the cohort of 157 infants who received fentanyl and doxapram simultaneously. The median gestational age of the fentanyl cohort was 27.1 weeks (range 24.3–31.2 weeks), median postnatal age at start of drug therapy was 4.5 days (range 0–68 days) and median body weight at start of drug therapy was 968 g (range 465–3,000 g). The median gestational age of the doxapram cohort was 26.1 weeks (range 24.0–29.4 weeks), median postnatal age at start of drug therapy was 17 days (range 1–52 days) and median body weight at start of drug therapy was 960 g (range 650–1,520 g).

Fentanyl was quantified in 370 samples from 92 patients, and doxapram and keto‐doxapram in 248 samples from 65 patients. For fentanyl, 78 (21%) of the 370 samples were measured below the LLOQ, and 19 (5%) below the LOD. For doxapram 29 (12%) and for keto‐doxapram, 33 (13%) of the 248 samples were below the LLOQ, and eight (3%) doxapram and six (2%) keto‐doxapram measurements were below the LOD. For doxapram, three (1.2%) samples were measured above the ULOQ.

## 4 DISCUSSION

We developed a robust UPLC–MS method for simultaneous quantification of fentanyl, sufentanil, cefazolin, doxapram and its active metabolite keto‐doxapram according to US Food and Drug Administration guidelines. The easy sample preparation, small required sample volume of 50 μL human plasma and short run time of 5.0 min perfectly met the objectives. We were able to analyze one plasma sample to simultaneously quantify multiple drugs that were part of one treatment, and combine samples with different drugs to be measured in one assay run.

Previously reported methods for quantification of these analytes concerned one of these analytes (with or without their metabolites) per assay or in a combination with other drugs. These combinations mostly concerned multiple drugs from the same drug class, i.e. sufentanil or fentanyl with other analgosedatives by Nosseir et al. (2014) and Fernandez Mdel et al. (2013), or cefazolin with beta‐lactams by Carlier et al. (2012) and Kirziazopoulos et al. (2017). Our assay concerned four drugs from three different anatomical therapeutic chemical classes: fentanyl and sufentanil as nervous system drugs; cefazolin as an anti‐infective drug; and doxapram as a respiratory drug. Herewith, our assay enables the quantification of four drugs in one sample simultaneously following one sample injection. This may be valuable for TDM as well as for research, concerning patients using a combination of these drugs as part of a particular treatment protocol. The burden to the patient may be reduced compared with a separate assay per drug, which is especially important concerning vulnerable (preterm) infants. Furthermore, samples with different drugs may be combined in one single run, which may improve the efficiency of the laboratory process.

In general, for all analytes, our assay performed better than or comparable to prior reported assays, even in comparison with assays measuring only a single analyte, which makes it easier to achieve good performance on run time, required sample volume and matrix effects (Aranda et al., 1988; Carlier et al., 2012; Clavijo et al., 2011; Fernandez Mdel et al., 2013; Hisada et al., 2013; Kiriazopoulos et al., 2017; LeGatt et al., 1986; Lillico et al., 2016; Mahlke et al., 2014; Nichol et al., 1980; Nosseir et al., 2014; Palleschi et al., 2003; Parker et al., 2016; Robson & Prescott, 1977; Saari et al., 2012; Suzuki et al., 2017). Furthermore, most assays use a different drug as an internal standard, whereas we used a deuterated form of fentanyl, which shows better comparable behavior to the analytes that are measured than using a different drug. Next, our sample preparation consisted of a simple one‐step protein precipitation method, whereas in most studies solid‐phase extraction is prescribed, or a liquid–liquid extraction with an evaporation and/or ultrafiltration step, or other additional steps.

For sufentanil, other reported assays required more plasma volume and a more complex sample preparation compared with our assay (Nosseir et al., 2014; Palleschi et al., 2003; Saari et al., 2012). Concerning doxapram, four of the five reported assays date from the 1990s (Aranda et al., 1988; LeGatt et al., 1986; Nichol et al., 1980; Robson & Prescott, 1977) and are inferior to our assay with respect to the use of a different drug for internal standard, sample preparation which requires an evaporation step, higher LLOQ, larger sample volume required, a longer run time and two assays not being able to measure keto‐doxapram. The recently published assay by Suzuki et al. (2017) required only a 25 μL plasma volume compared with our 50 μL, and an LLOQ for doxapram of 20 μg L ^−1^ compared with our 50 μg L^−1^. On the other hand, Suzuki et al. needed a 12 min run time and used propranolol as an internal standard, where we needed a 5 min run time and used deuterated fentanyl, and our sample preparation required fewer operational steps. Regarding cefazolin, multiple assays have been reported with comparable performance (Carlier et al., 2012; Kiriazopoulos et al., 2017; Lillico et al., 2016; Parker et al., 2016). The reported fentanyl assays required larger sample volumes except for Hisada et al. (2013), who needed only 20 μL and reached a lower LLOQ of 0.05 μg L^−1^ compared with our 0.1 μg L ^−1^.

Except for cefazolin and keto‐doxapram, the stability of all analytes was good, which means they were stable for at least 120 h when stored in the autosampler at 15°C. Cefazolin was only stable for 72 h and keto‐doxapram only for 48 h at 15°C. Suzuki et al. (2017) tested the stability of keto‐doxapram for 48 h at 10°C for autosampler conditions and also found it to be stable for that time. Stability during three cycles of freezing and thawing, and freezer stability studies of the analytes in the matrix are not presented because they were already carried out in previous published papers and do not depend on the analytical method. No relevant effects of freezing and thawing were found for all analytes, together with good stability at −20°C for 4 weeks (Clavijo et al., 2011; Kiriazopoulos et al., 2017; Mahlke et al., 2014; Nosseir et al., 2014; Palleschi et al., 2003; Parker et al., 2016; Saari et al., 2012; Suzuki et al., 2017).

Our assay fulfilled the desired criteria for accuracy, repeatability and reproducibility. Furthermore, for all analytes a good recovery was achieved and matrix effects were measured. These indicated the absence of interferences by matrix compounds, stable isotope‐labeled internal standard and co‐eluting compounds that may cause ion suppression and ion enhancement.

The ranges for linearity for all analytes were perfectly suitable for clinical pharmacology research, as well as for possible TDM purposes. The assay was successfully validated for clinical practice and research purposes for fentanyl and doxapram. Fentanyl was quantified in 370 plasma samples from 92 preterm infants, and doxapram and keto‐doxapram in 248 plasma samples from 65 preterms. The considerably high proportion of samples below the LLOQ (21% for fentanyl, 12% for doxapram, 13% for keto‐doxapram) was due to the objective of the study of investigating drug pharmacokinetics. Therefore, to estimate the clearance of the investigated drugs, opportunistic sample collection was allowed up to and beyond the time at which the plasma concentrations decreased below the LLOQ. All samples collected during continuous administration of both drugs were all above the LLOQ for all three analytes, and only above ULOQ for three doxapram samples shortly after a bolus administration. In conclusion, the assay performed well for samples in clinical practice. Furthermore, investigation is currently in progress in which this method has been applied to several pharmacokinetic studies in preterm infants up to elderly patients.

Despite the good performance, our assay has certain limitations. First, the stability of cefazolin and keto‐doxapram did not reach the desired 120 h at 15°C in the autosampler. However, as performance of the assay was finished within 48 h, this did not create a problem in practice. Second, although the plasma volume of 50 μL for performing the assay was small, this may be too much for some preterm infants, and for quantification of multiple drugs requiring the use of different assays. Third, the LLOQ of certain analytes in our assay was higher than some reported assays quantifying a single analyte. This is due to our goal of quantifying multiple analytes in one run simultaneously, which makes it more difficult to achieve maximal performance for all analytes. Nevertheless, the LLOQs of our assay all meet the clinically required limits of quantification. Fourth, the assay did not include inactive metabolites as these are not relevant for clinical practice. Therefore, only keto‐doxapram was included being the active metabolite of doxapram.

It has been suggested that TDM should be implemented as a supportive tool for analgosedation for fentanyl and sufentanil, which may help physicians increase patient comfort regarding intra‐ and inter‐operative interventions (Nosseir et al., 2014). The value of TDM has also been suggested for beta‐lactam antibiotics (Huttner, Harbarth, Hope, Lipman, & Roberts, 2015). Quantification of doxapram and keto‐doxapram during therapeutic dosages of doxapram may be relevant to improve successful therapy even further in the treatment of apnea of prematurity (Hayakawa et al., 1986) and for evaluation of safety (Barbe et al., 1999).

## 5 CONCLUSIONS

We have developed a method for the simultaneous quantification of fentanyl, sufentanil, cefazolin, doxapram and keto‐doxapram in 50 μL human plasma within a run time of only 5.0 min. This greatly facilitates further research into these drugs as well as possible TDM purposes, even in the smallest plasma volumes obtained from preterm infants.
